# PEGylation Effects on Amphiphilic Platinum(IV) Complexes: Influence on Uptake, Activation, and Cytotoxicity

**DOI:** 10.3390/pharmaceutics17040440

**Published:** 2025-03-29

**Authors:** Arpit Sharma, Md Al Amin, Man B. Kshetri, Suha Alqarni, Wjdan Jogadi, Jordan Solmen, Zexin Lin, Shirin Akter, Yao-Rong Zheng

**Affiliations:** 1 Department of Chemistry and Biochemistry, Kent State University, 236 Integrated Sciences Building, Kent, OH 44242, USA; 2 Department of Chemistry, University of Bisha, Bisha 67714, Saudi Arabia

**Keywords:** platinum(IV) prodrugs, PEGylation, cisplatin, mitochondria

## Abstract

**Background/Objectives:** The utilization of amphiphilic Pt(IV) complexes as prodrugs offers a promising strategy to revolutionize Pt-based cancer therapy by enhancing drug delivery and activation. While PEGylation is widely used to optimize drug properties, its impact on the biological behavior of amphiphilic Pt(IV) complexes remains unclear. This study systematically investigates how the PEGylation of varying molecular weights influences their cytotoxicity, cellular uptake, and activation. **Methods:** Pt(IV) complexes were synthesized with PEG chains of different molecular weights using HATU-catalyzed amide bond formation and copper-free click chemistry. Their biological properties were assessed through cell-based analyses, focusing on cytotoxicity, cellular uptake, and activation by biological reductants. **Results:** Small PEG modifications retained the potent cytotoxicity of amphiphilic Pt(IV) prodrugs, whereas large PEG chains significantly reduced efficacy. The decrease in potency was linked to impaired cellular uptake and mitochondrial accumulation. Additionally, large PEG modifications slowed the reduction and activation of Pt(IV) prodrugs by biological reductants, further limiting their anticancer activities. **Conclusions:** These findings underscore the critical role of PEGylation in metallodrug design and provide key insights into optimizing PEGylation strategies for enhancing platinum–based cancer therapies.

## 1. Introduction

A traditional foundation of cancer treatment has been platinum–based chemotherapy, with Pt(II) drugs like cisplatin, carboplatin, and oxaliplatin playing a crucial role in managing various cancers, including head and neck, testicular, ovarian, lung, and colorectal malignancies [1,2]. These drugs exert their effects by forming DNA cross–links, which inhibit cancer cell proliferation and induce cell death [3,4]. However, their clinical utility is often restricted by severe toxicities, such as kidney damage, neuropathy, and hearing loss [5,6]. Additionally, the growing challenge of drug resistance and subsequent cancer recurrence underscores the urgent need for innovative platinum–based therapies [7,8,9,10,11]. Exploring new strategies is essential to overcoming these limitations and enhancing treatment outcomes for cancer patients [12,13,14,15,16].

The use of kinetically “inert” Pt(IV) prodrugs with two accessible axial sites presents a promising strategy for developing novel Pt–based anticancer agents [1,17,18,19,20,21]. Extensive research has explored the chemical modifications of these axial ligands to unlock new potential in Pt drugs [18,22,23,24,25,26,27], including enhancing potency through synergy with secondary therapeutic agents [28,29], optimizing pharmacokinetics [29,30,31,32], improving tumortargeted delivery [13,17,33], harnessing immunological effects [34,35,36], and enabling stimuli-responsive release [22,24,37,38]. Notably, a unique class of Pt(IV) complexes, amphiphilic Pt(IV) complexes (AMPCs), has garnered significant attention in recent years [30,36,39,40,41,42]. This research was pioneered by the development of fatty acid–like Pt(IV) structures featuring axial ligands with long hydrophobic tails and carboxylate head groups, centered around a Pt(IV) core [30]. Recent studies indicate that the hydrophobic tails play a crucial role in protein binding, enhancing cellular uptake, stability, and activation mechanisms [30,40,41,42]. Meanwhile, the carboxylate head group serves as an excellent chemical handle for modifications, facilitating bioconjugation for targeted delivery or activation and enabling mechanisms of action distinct from conventional Pt(II) drugs [43,44]. The amphiphilic properties of these Pt(IV) complexes also promote self–assembly, making them ideal candidates for nanoformulation and encapsulation [45,46]. These innovative Pt(IV) prodrugs have demonstrated efficacy in both in vitro and in vivo studies [10,47]. However, further exploration is needed to fully understand their therapeutic potential and advance their clinical applications. PEGylation is a well–established approach for modifying and optimizing therapeutic molecules [48,49,50,51], yet its impact on amphiphilic Pt(IV) complexes remains poorly understood [52,53,54]. Investigating the effects of PEGylation on these novel structures could provide valuable insights into their pharmacological properties and therapeutic applications.

In this work, we systematically investigate how the PEGylation of varying molecular weights influences the biological properties of amphiphilic Pt(IV) complexes. Using HATU–catalyzed amide –bond formation and copper–free click chemistry [55,56,57], we synthesized Pt(IV) complexes with molecular weights ranging from low (<1000 Da) to high (>2000 Da) and conducted extensive cell–based analyses to assess their cytotoxicity, cellular uptake, and activation. Our findings reveal that small PEG modifications retain the potent cytotoxicity of amphiphilic Pt(IV) prodrugs while large PEG chains significantly reduce their efficacy by hindering cellular uptake and mitochondrial accumulation. Additionally, large PEG modifications significantly slow the reduction and activation of Pt(IV) prodrugs by biological reductants. These insights provide a deeper understanding of the role of PEGylation in metallodrug design, highlighting its potential to fine–tune therapeutic efficacy and cellular interactions.

## 2. Materials and Methods

**General information.** All the reagents were used without additional purification after being bought from Strem Chemicals (Newburyport, MA, USA), Aldrich (St. Louis, MO, USA), Broadpharm (San Diego, CA, USA), or Alfa Aesar (Haverhill, MA, USA). C16Pt was prepared using the reported literature [41,44]. Every reaction was conducted in typical atmospheric circumstances. The NMR data were acquired using a Bruker 400 NMR (Bruker Corporation, Billerica, MA, USA) (Frequency: 400 MHz for ^1^H NMR; 100 MHz for ^13^C NMR). The ^1^H and ^13^C{^1^H} NMR spectra’s chemical shifts were internally correlated with solvent signals (DMSO–d_6_ at δ = 2.50 ppm for ^1^H NMR and 40.45 ppm for ^13^C NMR). An Exactive Plus mass spectrometer (Thermo Scientific, Bremen, Germany) was used to record the high–resolution mass spectra of the produced ions. On an Agilent 1100 system, analytical HPLC was performed with C18 reverse–phase columns (Hypersil GOLD, 100 mm × 3 mm, 5 µm, Thermo Fischer Scientific, Waltham, MA, USA). Using a PerkinElmer PinAAcle 900Z spectrometer (PerkinElmer, Waltham, MA, USA), graphite furnace atomic absorption spectroscopy (GFAAS) experiments were performed. An Olympus IX70 (Olympus, Shijuku, Tokyo, Japan) inverted epifluorescence microscope with a digital CCD camera (QImaging, Surrey, BC, Canada) was used to capture the fluorescence images. ImageJ software (version 1.4.22, National Institute of Health, Bethesda, MD, USA) was used to analyze the images and quantify their intensities. The Invitrogen (Thermo Fisher Scientific, Waltham, MA, USA) LIVE/DEAD TM Cell Viability Kit (Cat. No. L3224) was used to perform the live/dead cell experiment. A BD Bioscience Accuri C6 flow cytometer (BD Biosciences, San Jose, CA, USA) was used to perform flow cytometry.

**Synthesis of compound 1**: C16Pt (50 mg, 0.071 mmol) and HATU (27 mg, 0.071 mmol) were taken into a vial, followed by the addition of 1 mL anhydrous DMF under a continuous stream of N_2_ gas. The mixture was stirred at room temperature (r.t.) for 20 min. mPEG-NH_2_ 250 (27.5 mg, 0.11 mmol) was then introduced to the reaction mixture, and after another 20 min of stirring in dark at room temperature, DIPEA (68 µL, 0.36 mmol) was added through a micro syringe. The reaction mixture was kept stirring overnight in dark at room temperature. Then, DMF was subsequently removed via rotary evaporation at an elevated temperature, and the residue was dissolved in a small amount of MeOH. Finally, purification was carried out using flash column chromatography. Yield: 43 mg (65%). ^1^H NMR (400 MHz, DMSO–d_6_): δ: 7.91 (t, *J* = 5.6 Hz, 1H), 6.51–6.60 (m, 7H), 3.50 (s, 17H), 3.20–3.09 (m, 4H), 2.86 (d, *J* = 6.8 Hz, 2H), 2.42 (t, *J* = 7.4 Hz, 2H), 2.27 (t, *J* = 7.4 Hz, 2H), 1.23 (s, 29H), 0.89–0.78 (t, *J* = 7.4 Hz, 3H). ^13^C NMR (100 MHz, DMSO–d_6_): δ: 179.98, 171.52, 163.94, 71.30, 69.81, 69.60, 69.11, 66.70, 58.08, 53.48, 41.76, 31.40, 31.33, 29.89, 29.10, 29.05, 28.96, 28.75, 26.51, 22.14, 18.09, 16.82, 14.00, 12.59. HR–MS (positive mode) for [C_32_H_69_Cl_2_N_4_O_10_Pt]^+^: *m*/*z* calculated: 934.4036, observed: 934.4043. Purity: 97% determined using HPLC.

**Synthesis of compound 2:** C16Pt (50 mg, 0.071 mmol) and HATU (27 mg, 0.071 mmol) were taken into a vial, followed by the addition of 1 mL anhydrous DMF under a continuous stream of N_2_ gas. The mixture was stirred at room temperature (r.t.) for 20 min. mPEG-NH_2_ 1K (110 mg, 0.11 mmol) was then introduced to the reaction mixture, and after another 20 min of stirring at room temperature, DIPEA (68 µL, 0.36 mmol) was added through a micro syringe. The reaction mixture was kept stirring overnight in the dark at room temperature. Then, DMF was subsequently removed via rotary evaporation at an elevated temperature, and the residue was dissolved in a small amount of MeOH. Finally, purification was carried out using flash column chromatography. Yield: 67 mg (63%). ^1^H NMR (400 MHz, DMSO–d_6_): δ: 7.81 (t, *J* = 5.6 Hz, 1H), 6.61–6.52 (m, 7H), 3.50 (s, 89H), 3.43–3.42 (m, 2H), 3.23 (s, 3H), 3.06 (m, 2H), 2.89–2.86 (m, 2H), 2.43 (t, *J* = 7.4 Hz, 2H), 2.25 (t, *J* = 7.4 Hz, 2H), 1.23 (s, 31H), 0.88–0.82 (t, 3H).δ: ^13^C NMR (100 MHz, DMSO–d_6_): δ: 179.99, 171.27, 163.92, 71.29, 69.79, 69.60, 69.55, 68.10, 58.07, 35.83, 31.47, 31.32, 29.88, 29.33, 29.09, 29.03, 28.94, 28.73, 26.50, 22.12, 13.99. HR–MS (positive mode) for [C_65_H_136_Cl_2_N_4_O_26_Pt]^2+^: *m*/*z* calculated: 827.4232, observed: 827.4229. Purity: 98% determined using HPLC.

**Synthesis of compound 3:** C16Pt (50 mg, 0.071 mmol) and HATU (27 mg, 0.071 mmol) were taken into a vial, followed by the addition of 1 mL anhydrous DMF under a continuous stream of N_2_ gas. The mixture was stirred for 20 min at room temperature (r.t.). mPEG-NH_2_ 2K (220 mg, 0.11 mmol) was then introduced to the reaction mixture, and after another 20 min of stirring at room temperature, DIPEA (68 µL, 0.36 mmol) was added through a micro syringe. The reaction mixture was kept stirring overnight in the dark at room temperature. Then, DMF was subsequently removed via rotary evaporation at an elevated temperature, and the residue was dissolved in a small amount of MeOH. Finally, purification was carried out using flash column chromatography. Yield: 94 mg (56%). ^1^H NMR (400 MHz, DMSO–d_6_): δ: 7.90 (t, *J* = 5.6 Hz, 1H), 6.56 (m, 7H), 3.50 (m, 185H), 3.43–3.40 (m, 2H), 3.23 (s, 3H), 3.20–3.15 (m, 2H), 2.91–2.83 (m, 2H), 2.43 (t, *J* = 7.4 Hz, 2H), 2.27 (t, *J* = 7.4 Hz, 2H), 1.23 (s, 33H), 0.87–0.83 (t, 3H).^13^C NMR (100 MHz, DMSO–d_6_): δ: 179.99, 171.52, 163.93, 77.40, 72.31, 71.30, 69.11, 62.20, 60.22, 58.07, 58.05, 53.55, 31.39, 31.33, 30.72, 29.89, 29.14, 29.12, 29.10, 29.05, 28.96, 28.75, 27.01, 22.16, 18.09, 16.75, 13.97. HR–MS (positive mode) for [C_108_H_224_Cl_2_N_4_O_48_Pt]^4+^: *m*/*z* calculated: 653.1059, observed: 653.1057. Purity: 96% determined via HPLC.

**Synthesis of C16Pt**–**DBCO:** C16Pt (100 mg, 0.138 mmol) and HATU (65 mg, 0.171 mmol) were taken into a vial, followed by the addition of 1 mL anhydrous DMF under a continuous stream of N_2_ gas. The mixture was stirred at room temperature (r.t.) for 20 min, and DBCO Amine (107.75 mg, 0.39 mmol) was then introduced to the reaction mixture.and after another 20 min of stirring at room temperature, DIPEA (105 µL, 0.6 mmol) was added through a micro syringe. The reaction mixture was kept stirring overnight in the dark at room temperature. Then, DMF was subsequently removed via rotary evaporation at an elevated temperature, and small amount of MeOH was used to dissolve the residue. Finally, purification was carried out using flash column chromatography. Yield: 72.9 mg (54%). ^1^H NMR (400 MHz, DMSO–d): δ 7.66 (t, *J* = 5.6 Hz,1H), 7.62 (m, 1H), 7.46 (m, 4H), 7.38 (m, 3H), 6.60–6.51 (m, 7H), 5.02 (d, *J* = 14.0 Hz, 1H), 3.64 (d, *J* = 14.0 Hz, 1H), 2.91–2.89 (m, 4H), 2.42 (m, 1H), 2.33 (t, *J* = 7.4 Hz, 2H), 2.12 (t, *J* = 7.5 Hz, 2H), 1.82 (m, 1H), 1.23 (s, 28H), 0.85 (t, *J* = 7.4 Hz, 3H). ^13^CNMR (100 MHz, DMSO–d_6_): δ 177.90, 171.97, 170.20, 151.43, 148.41, 132.41, 129.55, 128.92, 128.22, 128.09, 127.74, 126.82, 125.19, 122.49, 121.45, 114.31, 108.07, 64.96, 54.84, 35.21, 34.90, 34.29, 31.33, 29.08, 29.04, 28.94, 28.77, 28.75, 28.61, 25.14, 22.13, 15.20, 13.99. HR–MS (positive mode) for [C_39_H_59_Cl_2_N_5_O_6_Pt+H]^+^: *m*/*z* calculated: 959.3563, observed: 960.3561. Purity: 97% determined using HPLC.

**Synthesis of compound 4:** C16Pt–DBCO and Methyl–PEG–8–Azide were mixed in a 1:1 ratio in DMSO and were allowed to react via copper-free click chemistry at room temperature. After 2 h, semi–preparative HPLC was used to purify the reaction mixture and the fractions that contained the desired product were isolated which was lyophilized to obtain the final products. This compound was confirmed using HPLC & HR–ESI–MS. HR–MS (positive mode) for [C_56_H_94_C_l2_N_8_O_14_Pt]^2+^: *m*/*z* calculated: 685.3038, observed: 685.3030.

**Synthesis of compounds **5** and **6**:** C16Pt–DBCO and Methoxy–PEG–Azide–5K, Methoxy–PEG–Azide–20K were mixed in a 1:1 ratio in DMSO and were allowed to react via copper–free click chemistry at r.t After 2 h, semi–preparative HPLC was used to purify the reaction mixture and the fractions that contained the desired product were isolated which was lyophilized to obtain the final products. These complexes were confirmed by HPLC.

**Stability analysis of complexes against biological reductant:** The stability of compound **4**–**6** was studied in the presence of ascorbic acid (200 µM) and glutathione (1 mM) over the period of 24 h as shown in Figure 5. All the compounds were dissolved in PBS along with ascorbic acid or glutathione such that the final Pt concentration is 100 µM and the final volume was 500 µL. Initial Pt concentration was measured using GFAAS and was considered as 0 h, solution of compound **4** was transferred to 1 kD dialysis bag whereas compound **5**,**6** were kept in 3.5 kD dialysis bags. Each bag was kept in a big container containing 1x PBS and was stirred for 24 h. GFAAS was used to determine the Pt concentration.

**Cell culture:** A2780cis and A2780 cell lines were bought from SigmaAldrich and cultivated in RPMI 1640 containing 1% Penicillin–Streptomycin (Corning NY, USA) and 10% FBS (Atlanta Biologicals, Flowery, Branch, GA, USA) along with L–glutamine (Corning, NY, USA). The American Type Culture Collection provided the Hela cell line and MDA–MB–231 cell line, which were then cultured in DMEM containing L–glutamine, 1 g/L glucose, and sodium pyruvate (Corning), with 1% Penicillin–Streptomycin (Corning) and 10% FBS as supplements. Every cell line was grown in an environment with 5% CO_2_ at 37 °C. Trypsinization was used to pass cells after they reached ~90% confluence, and they were passaged in a 1:5 ratio.

**MTT cell viability assays.** MTT assays were used to evaluate the cytotoxic potency of cisplatin and compounds **1**–**6** against A2780cis, A2780, Hela and MDA–MB–231 cell lines. 96–well plates were seeded with 100 μL solution of DMEM or RPMI media containing 4 × 10^4^ cells/mL. The plates were then incubated for 24 h at 37 °C with 5% CO_2_ to promote cell adhesion. Subsequently, 50 μL of DMEM or RPMI medium containing different concentrations of the test compounds and cisplatin were added to each well and then incubated for 48 h. After this incubation period, 30 μL of MTT solution (3–(4,5–dimethylthiazol–2–yl)–2,5-diphenyltetrazolium bromide, 5.0 mg/mL in PBS, Alfa Aesar) was added to each well and incubated for 3–4 h. After aspiration of the medium the purple formazan crystals were dissolved upon addition of 200 μL of DMSO. The plates were gently shaken at room temperature for 10 min, and the 562 nm absorbance was measured using a BioTek ELx800 plate reader (Bio Tek Instruments, Winooski, VT, USA). Origin software (Origin 2022b, 9.95) was used for data analysis to generate dose-response curves and determine IC_50_ values. All experiments were conducted in triplicate. 

**LIVE/DEAD cell viability assay**. compound **1**–**6** and cisplatin’s effectiveness was assessed in vitro in A2780cis ovarian cancer cells using LIVE/DEAD cell viability assay. In a 35 mm sterile culture dishes (Celltreat Scientific Products) A2780cis cells were seeded for 24 h in an RPMI medium supplemented with 1% penicillin/streptomycin and 10% FBS. Each compound (1 µM) was added. The dishes were then incubated for 48 h at 37 °C in a 5% CO_2_ at-mosphere. Following incubation, the dishes were washed twice with 1 mL PBS and once with 1 mL dye–free RPMI medium to eliminate serum esterase activity from the serum-supplemented medium. A LIVE/DEAD working solution was prepared by mixing 5 µL calcein AM (4 mM in anhydrous DMSO) and 10 µL ethidium homodimer-1 (2 mM in DMSO/water, 1:4 *v*/*v*) into 10 mL dye–free RPMI. Next, 2 mL of this solution was added to each culture dish and incubated for 30 min at room temperature. Finally, images were captured using fluorescence microscopy after replacing the medium with 1 mL dye–free RPMI. 

**GFAAS analysis of cellular platinum contents in A2780cis cells.** A2780cis cells were cultured in a 6–well plate at a density of 5 × 10^5^ cells in each well, and incubated at 37 °C with 5% CO_2_ for 24 h to promote cell adherence. The following day, the cells were exposed to cisplatin ([Pt] = 2 μM) and compounds **4**–**6** ([Pt] = 2 μM) and were incubated for 24 h at 37 °C and 5% CO_2_. The cells were then collected via trypsinization (1 mL), washed with PBS (1 mL), and tallied. Next, the cells were digested overnight at room temperature in 200 μL of 65% HNO_3_. Using GFAAS, the Pt contents in the cells were examined. Every experiment was run in triplicate.

**Measurements of mitochondrial platinum contents in A2780cis cells**. A2780cis cells were cultured in a 6–well plate at a density of 5 × 10^5^ cells in each well and incubated at 37 °C with 5% CO_2_ for 24 h to promote cell adherence. The following day, the cells were exposed to cisplatin ([Pt] = 2 μM) and compounds **4**–**6** ([Pt] = 2 μM) and were incubated for 24 h at 37 °C and 5% CO_2_. The cells were then collected by trypsinization (1 mL), washed with PBS (1 mL), and tallied. Thermo ScientificTM Mitochondria Isolation Kit (Waltham, MA, USA) for Mammalian Cells was utilized to isolate mitochondrial fractions. After dissolving the mitochondrial fraction in 200 µL of 65% HNO_3_, the mixture was mixed at 400 rpm on an Eppendorf ThermoMixerTM F1.5 at room temperature overnight. The fractions were then diluted four times in water, and GFAAS was used to determine the platinum content. Every experiment was triplicated.

**Flow cytometric analysis of DNA damage using γH2AX**. A2780cis cells were seeded in a 6–well plate at a density of 1 × 10^5^ cells per well and incubated for 24 h at 37 °C in a 5% CO_2_ atmosphere. Following incubation, the cells were treated with cisplatin (10 µM) and compounds **4** and **5** (0.2 µM each), while one well in the plate served as a control. The plates were then incubated for 48 h at 37 °C in a 5% CO_2_ atmosphere. After incubation, live cells were collected via trypsinization, washed twice with 1 mL PBS, and resuspended in 250 µL BD permeabilization solution followed by incubation at 4 °C for 20 min. The cell pellet was collected via centrifugation and washed with 1 mL of 1× washing buffer. Next, 5 µL of Alexa Fluor 488–conjugated anti–H2AX antibody was added to 50 µL of the resuspended cell pellet and incubated for 60 min in the dark at room temperature for 60 min. After incubation, the cells were centrifuged at 1400 rpm for 5 min and resuspended in 300 µL PBS. The PBS cell suspension was analyzed using the FITC (FL1) channel on a BD Accuri C6 flow cytometer (BD Biosciences, Franklin Lakes, NJ, USA).

**Flow cytometric analysis of MitoSOX**. In 6–wells plate, A2780cis cells were seeded at a density of 1 × 10^5^ cells per well and incubated for 24 h at 37 °C in a 5% CO_2_ atmosphere. Following incubation, the cells were treated with cisplatin ([Pt] = 10 μM) or Compound **4**, and **5** ([Pt] = 0.2 μM each) while one well in the plate served as a control. The plates were then incubated at 37 °C in a 5% CO_2_ atmosphere for 48 h. After aspirating the medium, 1 mL of PBS was used to wash the cells. The cells were then incubated for 60 min at 37 °C with 5% CO_2_ in the dark using 5 μM of MitoSOX reagent in fresh media. Trypsinized cells were gathered. PBS was used to wash the cell pellet twice. After being re-dissolved into PBS with 0.5% BSA to reach 1 × 10^6^ cells/mL, the cells were examined using a BD Accuri C6 flow cytometer (BD Biosciences, San Jose, CA, USA) with a FL–2 channel, and FlowJo, version 10.8 (FlowJo LLC, Arshland, OR, USA) was used to process the data.

## 3. Results and Discussion

**Figure 1:**
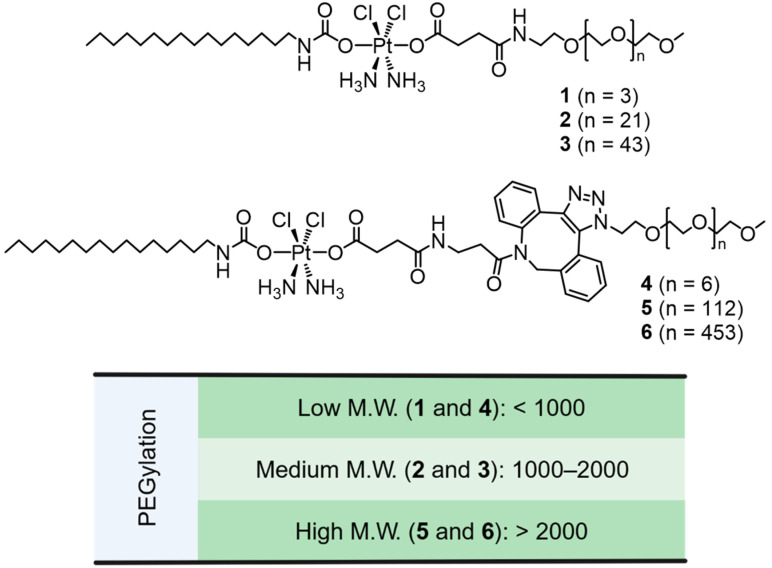
Structures of amphiphilic Pt(IV) complexes (AMPCs) with PEGylation ranging from a low to high molecular weight (M.W.).

**Synthesis and Characterization of Amphiphilic Pt(IV) Complexes (AMPCs) with PEG Groups**. AMPCs (**1**–**6**) were synthesized via two approaches: (1) HATU–catalyzed amide bond formation (Figure S1) and (2) copper–free click chemistry (Figures S2 and S7). In the first approach, amino moieties with a small to medium molecular weight (M.W.) PEG groups (Figure 1) were conjugated to C16Pt through an HATU–catalyzed amide bond formation, yielding final compounds (**1**–**3**) purified by flash column chromatography (56–65% yield). In the second approach, Pt(IV) prodrugs (**4**–**6**) were synthesized via copper-free click reactions between C16Pt–DBCO and corresponding azido moieties with small and large M.W. PEG groups (Figure 1). The Pt(IV) complexes (**1**–**3** and C16Pt–DBCO) were characterized using ^1^H and ^13^C NMR spectroscopy, HPLC, and electrospray ionization mass spectrometry (ESI–MS) (Supporting Information, Figures S3–S7). In the ^1^H NMR spectra, a broad signal at ~6.6 ppm corresponds to the Pt(IV) amine groups, while the ~2.8 ppm signal is attributed to the CH_2_ group adjacent to the carbamate. Signals at 6.8–8.6 ppm correspond to the amide functional groups. ESI–MS analysis confirmed isotopically resolved signals matching theoretical values, and HPLC analysis verified the high purity of the final compounds.

**Cytotoxicity Profiles of AMPCs with Varying PEGylation.** Amphiphilic Pt(IV) prodrugs undergo intracellular reduction, releasing cisplatin, which induces mitochondrial and nuclear DNA damage [41]. In vitro anticancer efficacy of these AMPCs (**1**–**6**) against four human cancer cell lines: A2780cis, A2780, HeLa, and MDA–MB–231, was evaluated using MTT assay. A2780cis represents a platinum–resistant ovarian cancer model, while MDA–MB–231 is a triple–negative breast cancer subtype known for its treatment challenges.

**Figure 2:**
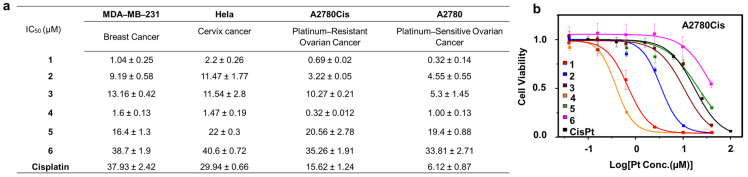
Cytotoxicity profiles of amphiphilic Pt(IV) complexes with varying PEGylation: (**a**) Table summarizing IC_50_ values of Pt compounds against human cancer cell lines. (**b**) Dose–response (killing) curves of compounds **1**–**6** in A2780cis cells over 48 h.

Cells were treated with cisplatin or AMPCs (**1**–**6**) for 48 h, and IC_50_ values (concentration required to inhibit 50% cell growth) were determined (Figure 2a). Compounds **1** and **4**, with small PEG modifications, exhibited significantly lower IC_50_ values than compounds **2**, **3**, **5**, and **6**, as well as cisplatin across all cell lines. Notably, in A2780cis, compound **4** (IC_50_ = 0.32 ± 0.012 μM) was up to 49 times more potent than cisplatin (IC_50_ = 15.62 ± 1.24 μM), highlighting its enhanced efficacy.

**Figure 3:**
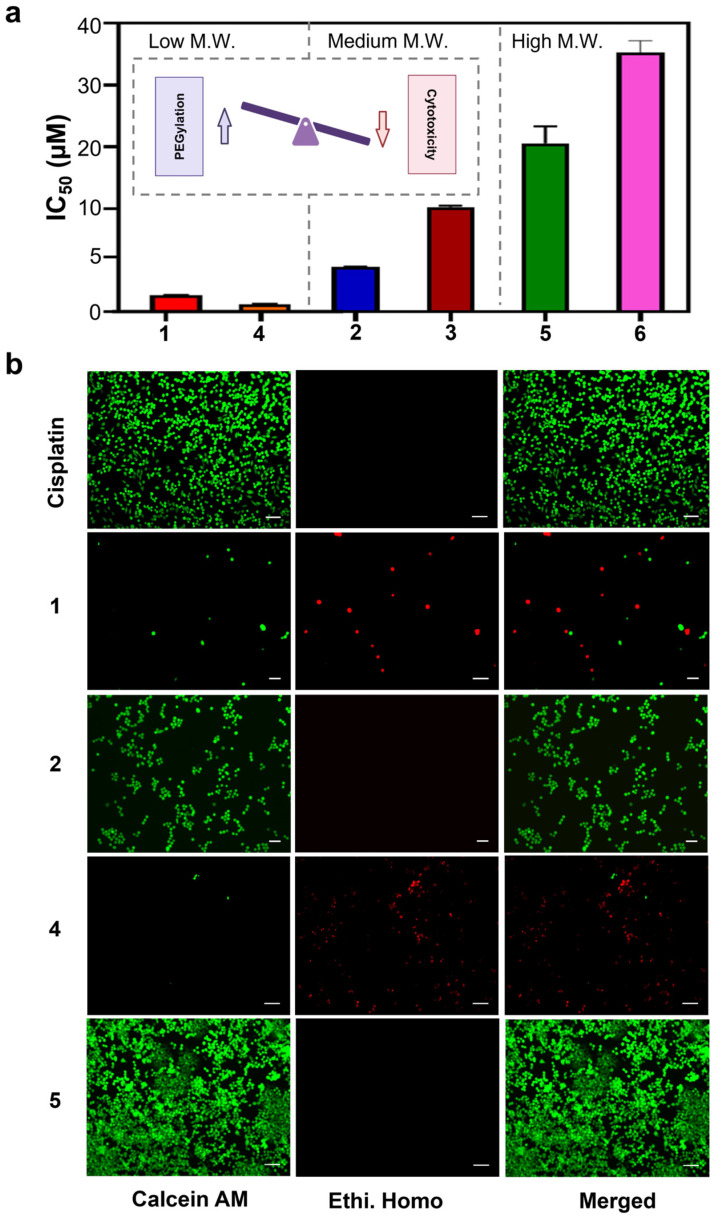
Impact of PEGylation on the cytotoxicity of amphiphilic Pt(IV) complexes: (**a**) Bar graph comparing IC_50_ values of AMPCs (**1**–**6**) with varying PEG modifications in A2780cis cells. (**b**) Live/dead cell assay images of A2780cis cells treated with compounds **1**, **2**, **4**, and **5** ([Pt] = 1 µM) for 48 h. Scale bar: 100 µm.

However, PEG chain length influenced cytotoxicity in a non–uniform manner. While small and medium PEG modifications generally improved cytotoxic profiles compared to cisplatin, large PEG chains (e.g., 5k and 20k) drastically reduced potency, yielding higher IC_50_ values than cisplatin (Figure 3a). To further validate this trend, live/dead cell viability assays were performed (Figure 3b). Compounds **1** and **4** effectively eliminated A2780cis cells, while compound **2** (medium PEG) significantly reduced cell population, and compound **5** (large PEG) failed to induce cell death at 1 µM after 48 h of incubation.

Overall, a clear trend emerged: increasing PEG molecular weight decreases cytotoxicity. While small and medium PEG modifications outperform cisplatin, large PEGylation significantly reduces activity. These findings underscore the potential of head group modifications with PEG chains to fine-tune the therapeutic effects of this class of metallodrugs, offering a promising strategy for optimizing their anticancer activity.

**Figure 4:**
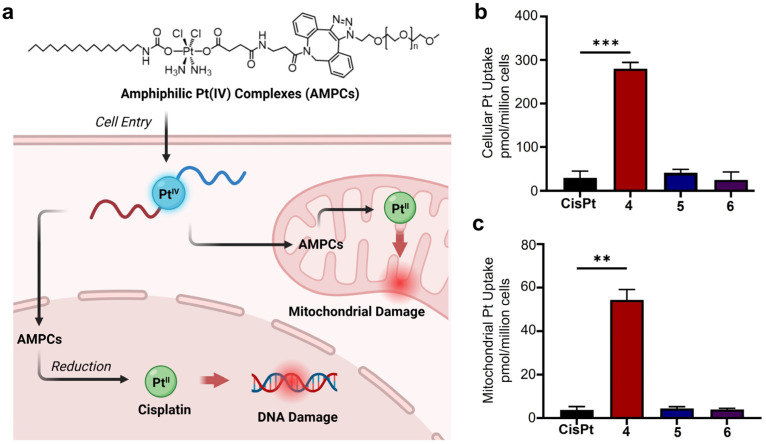
Impact of PEGylation on cellular uptake and mitochondrial accumulation of amphiphilic Pt(IV) complexes: (**a**) Schematic representation of the mechanism of action of AMPCs. (**b**) Cellular uptake and (**c**) mitochondrial accumulation of AMPCs (4, 5, 6) and cisplatin (CisPt) in A2780cis cells after 48 h. Statistical analysis was performed using a *t*-test, with *p*-values indicating significance (*p* < 0.01 (**), *p* < 0.001 (***)).

**Cellular Uptake and Mitochondrial Accumulation of AMPCs with Varying PEG Lengths.** To investigate the impact of PEG chain length on anticancer activity, we examined cellular uptake and mitochondrial accumulation (Figure 4). Cellular uptake, a key factor in metallodrug efficacy, was quantified using graphite furnace atomic absorption spectroscopy (GFAAS) in cells treated with compounds **4**–**6** and cisplatin. As shown in Figure 4b, compound **4** (short PEG chain, PEG_400_) exhibited over 11–fold higher uptake (280 ± 14.51 pmol Pt/million cells) compared to compound **6** (longest PEG chain, PEG_20k_; 25 ± 18 pmol Pt/million cells) and over nine–fold greater uptake than cisplatin (30 ± 15 pmol Pt/million cells). Similarly, mitochondrial accumulation was assessed, revealing that compound **4** had 14–fold higher mitochondrial Pt content (54.33 ± 4.784 pmol Pt/million cells) than compound **6** (3.95 ± 0.62 pmol Pt/million cells) and 14–fold greater accumulation than cisplatin (3.82 ± 1.49 pmol Pt/million cells) (Figure 4c). These findings demonstrate that small PEG modifications enhance cytotoxicity by promoting cellular uptake and mitochondrial accumulation, whereas large PEG chains significantly reduce efficacy by hindering both processes, emphasizing the importance of PEG length in optimizing the therapeutic potential of these metallodrugs.

**Figure 5:**
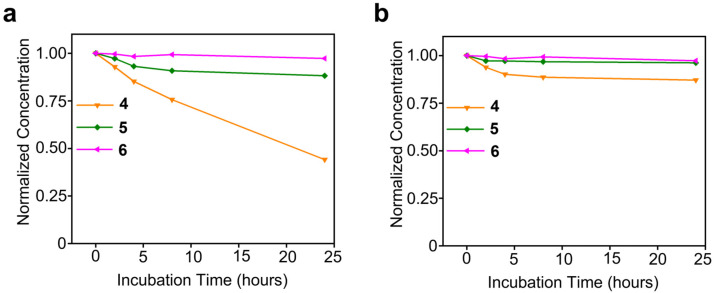
**Impact of PEGylation on reduction in amphiphilic Pt(IV) complexes**: (**a**) Stability curves of compounds **4**–**6** in ascorbic acid (200 µM). (**b**) Stability curves of compounds **4**–**6** in glutathione (1 mM).

**Reduction in AMPCs with Varying PEGylation.** To assess the impact of PEG chain length on the activation of Pt(IV) prodrugs, we examined the reduction in AMPCs by biological reductants, namely, ascorbic acid and glutathione. The stability of compounds **4**–**6** was evaluated in the presence of ascorbic acid (200 µM) and glutathione (1 mM) over 24 h, as shown in Figure 5. All compounds were dissolved in PBS containing one of the reducing agents, maintaining a final Pt concentration of 100 µM.

The initial Pt concentration was measured using GFAAS as the 0h reference point. Compound **4** was placed in a 1 kDa dialysis membrane, while compounds **5** and **6** were placed in 3.5 kDa membranes. Each dialysis bag was immersed in a stirred PBS reservoir for 24 h, after which Pt concentrations were remeasured using GFAAS.

The dialysis study revealed that compound **6** exhibited the highest stability, while compound **4** showed the lowest stability and the greatest tendency for reduction/activation among the tested compounds. These findings demonstrate that PEGylation significantly enhances stability, with longer PEG chains providing greater resistance to reduction, thereby stabilizing AMPCs and inhibiting their activation. This effect likely correlates with the lower cytotoxicity profiles observed in the previous section.

**Cellular Responses of AMPCs with Varying PEG Head Groups**. Building on the observation that shorter PEG chains enhance intracellular Pt accumulation, we hypothesized that such modifications would lead to greater mitochondrial and DNA damage, whereas larger PEG chains would mitigate these effects. To test this, we assessed γH2AX levels as a marker of DNA damage and used MitoSOX to measure mitochondrial ROS levels.

**Figure 6:**
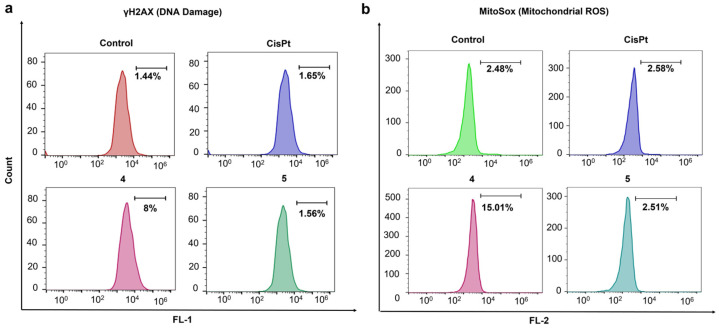
**Impact of PEGylation on cellular responses of amphiphilic Pt(IV) complexes**: (**a**) Flow cytometric analysis of γH2AX in A2780cis cells treated with AMPCs (**4** or **5**) or cisplatin for 48 h. (**b**) Flow cytometric analysis of MitoSOX in A2780cis cells treated with AMPCs (**4** or **5**) or cisplatin for 48 h.

Flow cytometric analysis was conducted to examine mitochondrial ROS and γH2AX levels in A2780cis cells treated with compounds **4**, **5**, and cisplatin. As shown in Figure 1, compound **4** has a short PEG chain, whereas compound **5** features a long PEG chain. According to the flow cytometry results (Figure 6a), treatment with compound **4** induced significant DNA damage, as indicated by elevated γH2AX levels compared to the control and cisplatin. In contrast, treatment with compound **5** resulted in minimal DNA damage, highlighting the impact of PEG length.

Similarly, mitochondrial ROS levels were significantly higher in cells treated with compound **4** compared to the control or compound **5** (Figure 6b). Notably, cisplatin induced only a slight increase in mitochondrial ROS levels, consistent with its known mechanism of action.

These findings suggest that compound **4**, with a short PEG chain (PEG_400_), effectively induces mitochondrial and DNA damage, whereas compound **5**, with a long PEG chain (PEG_5000_), exhibits reduced cytotoxicity. This difference may be attributed to encapsulation effects, where larger PEG modifications enhance stability against external reducing agents, thereby limiting Pt activation and cellular damage.

## 4. Conclusions

This study presents the first comprehensive investigation into the effects of PEGylation on the biological properties of a novel class of Pt–based prodrugs, namely, amphiphilic Pt(IV) complexes (AMPCs). We synthesized a small library of AMPCs with varying PEG chain lengths as head groups and discovered that these modifications significantly influence cytotoxicity, ranging from highly potent (PEG_250_) to least potent (PEG_20k_). Notably, lowmolecularweight PEG chains (PEG_250_ and PEG_400_) enhance potency, whereas longer PEG chains significantly reduce cytotoxicity.

To elucidate the role of PEG length in cytotoxicity, we examined cellular uptake and mitochondrial accumulation and cellular uptake using GFAAS, as well as DNA and mitochondrial and DNA damage via flow cytometry. Our findings reveal that AMPCs with short PEG chains efficiently penetrate cancer cells and accumulate in mitochondria, inducing strong cellular responses, including nuclear DNA damage and mitochondrial ROS generation, ultimately leading to effective cancer cell eradication. In contrast, AMPCs with long PEG chains exhibit significantly lower uptake and weaker cellular responses.

A particularly intriguing observation is that larger PEG modifications (PEG5k and PEG20k) significantly impair reduction through biological reductants like ascorbic acid, consequently rendering these compounds inactive. This finding provides valuable insight into metallodrug design and underscores the importance of carefully selecting PEG length to optimize therapeutic efficacy.

Overall, our study provides valuable insights into the structural optimization of Pt–based prodrugs, emphasizing the potential of PEGylation as a tunable strategy for developing more potent and selective anticancer therapeutics. This work lays a solid foundation for future research focused on engineering Pt compounds with tailored PEG chains, with the goal of enhancing therapeutic efficacy while minimizing side effects.

## Data Availability

Data are contained within the article and Supplementary Materials.

## Supplementary Materials

The following supporting information can be downloaded at: https://www.mdpi.com/article/10.3390/pharmaceutics17040440/s1. Figure S1: Synthetic route for preparing Amphiphilic Pt(IV) Complexes 1–3; Figure S2: Synthetic route for preparing C16Pt–DBCO; Figure S3: Characterization of compound **1**: A. ^1^H NMR spectrum in DMSO–d_6_; B. ^13^C NMR spectrum in DMSO–d_6_; C. HPLC analysis; D. High resolution ESI–MS spectra; Figure S4: Characterization of compound **2**: A. ^1^H NMR spectrum in DMSO–d_6_; B. ^13^C NMR spectrum in DMSO–d_6_; C. HPLC analysis; D. High resolution ESI–MS spectra; Figure S5: Characterization of compound **3**: A. ^1^H NMR spectrum in DMSO–d_6_; B. ^13^C NMR spectrum in DMSO–d_6_; C. HPLC analysis; D. High resolution ESI–MS spectra; Figure S6: Characterization of compound C16Pt–DBCO: A. ^1^H NMR spectrum in DMSO–d_6_; B. ^13^C NMR spectrum in DMSO–d_6_; C. HPLC analysis; D. High resolution ESI-MS spectra; Figure S7: Characterization of compound **4**–**6**: A. Formation of Complex 4–6 via copper-free click reaction between C16Pt–DBCO and mPEG–Azides; B. HPLC Analysis of C16Pt–DBCO and 4–6; C. High resolution ESI–MS spectrum of compound **4**; Figure S8: Killing curves of compounds **1**–**6** and cisplatin (CisPt) incubated for 48 h using A2780cis, A2780, MDA–MB–231, and Hela cells; Figure S9:Fluorescence microscope images of Live/Dead assay for A2780cis cells treated with (Control), **1**–**6**, (1 µM, 48 h) and cisplatin (100 µM) at 37 °C under 5% CO_2_. Scale bar = 100 µm
