# Supplementary material for: PEGylation Effects on Amphiphilic Platinum(IV) Complexes: Influence on Uptake, Activation, and Cytotoxicity
Source: Pharmaceutics. 2025 Mar 29;17(4):440. doi: 10.3390/pharmaceutics17040440 (PMC12030465; doi:10.3390/pharmaceutics17040440)
Supplement: Supplementary file 1 [file pharmaceutics-17-00440-s001.zip › pharmaceutics-3536888-supplementary.pdf]

Supporting Information

PEGylation Effects on Amphiphilic Platinum(IV) Complexes: Influence on Uptake, Activation, and Cytotoxicity

Arpit Sharma [1†], Md Al Amin [1†], Man B. Kshetri [1], Suha Alqarni [1,2], Wjdan Jogadi [1], Jordan Solmen [1], Zexin Lin [1], Shirin Akter [1], and Yao-Rong Zheng [1,*]

1. Department of Chemistry and Biochemistry, Kent State University, 236 Integrated Sciences Building, Kent, OH 44242, USA
2. Department of Chemistry, University of Bisha, Bisha 67714, Saudi Arabia

† These authors contributed equally to this work

* Correspondence: yzheng7@kent.edu; Tel.: +1-330-672-2267

Table of Contents

Figure S1. Synthetic route for preparing complexes 1–3 2

Figure S2. Synthetic route for preparing C16Pt–DBCO 3

Figure S3. Characterization of compound 1 4

Figure S4. Characterization of compound 2 5

Figure S5. Characterization of Compound 3 6

Figure S6. Characterization of Compound C16Pt–DBCO 7

Figure S7. Characterization of Compound 4–6 8

Figure S8. Cell viability curve of compounds 1–6 & cisplatin 9

Figure S9. Fluorescence microscope images of Live/Dead assay for A2780cis cells treated with PBS (Control), 1–6, and Cisplatin 10

Supporting Information

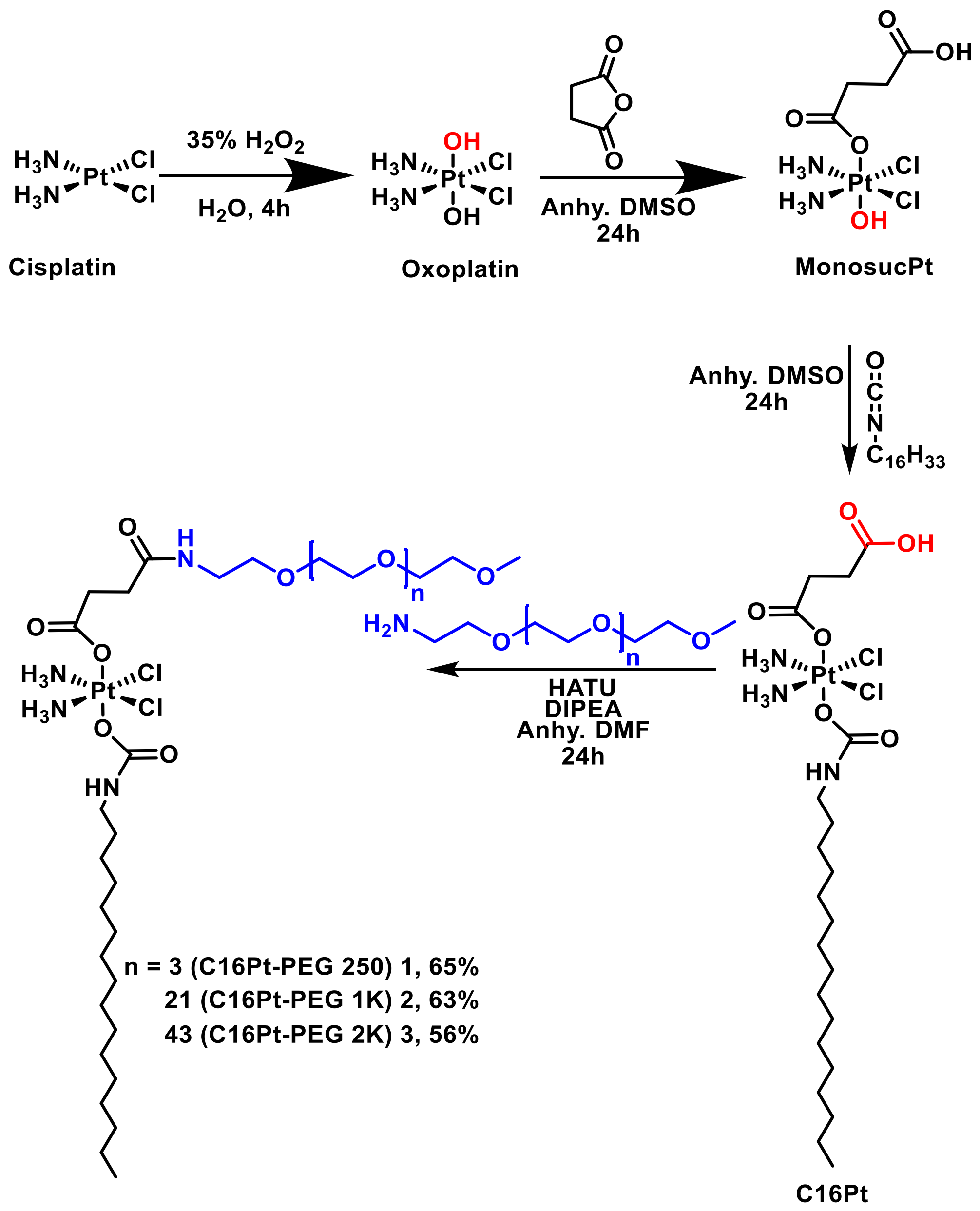

Figure S1. Synthetic route for preparing Amphiphilic Pt(IV) Complexes **1–3.**

Supporting Information

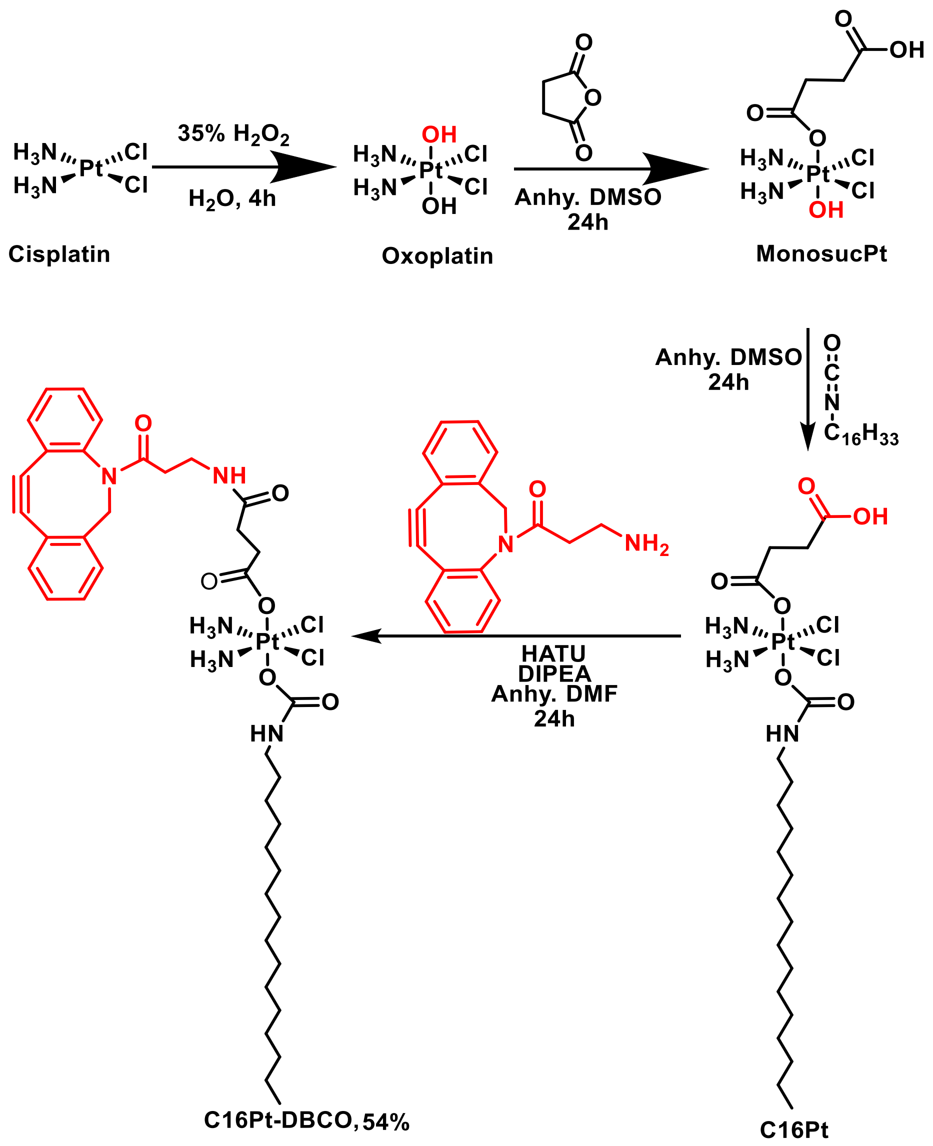

Figure S2. Synthetic route for preparing **C16Pt–DBCO.**

Supporting Information

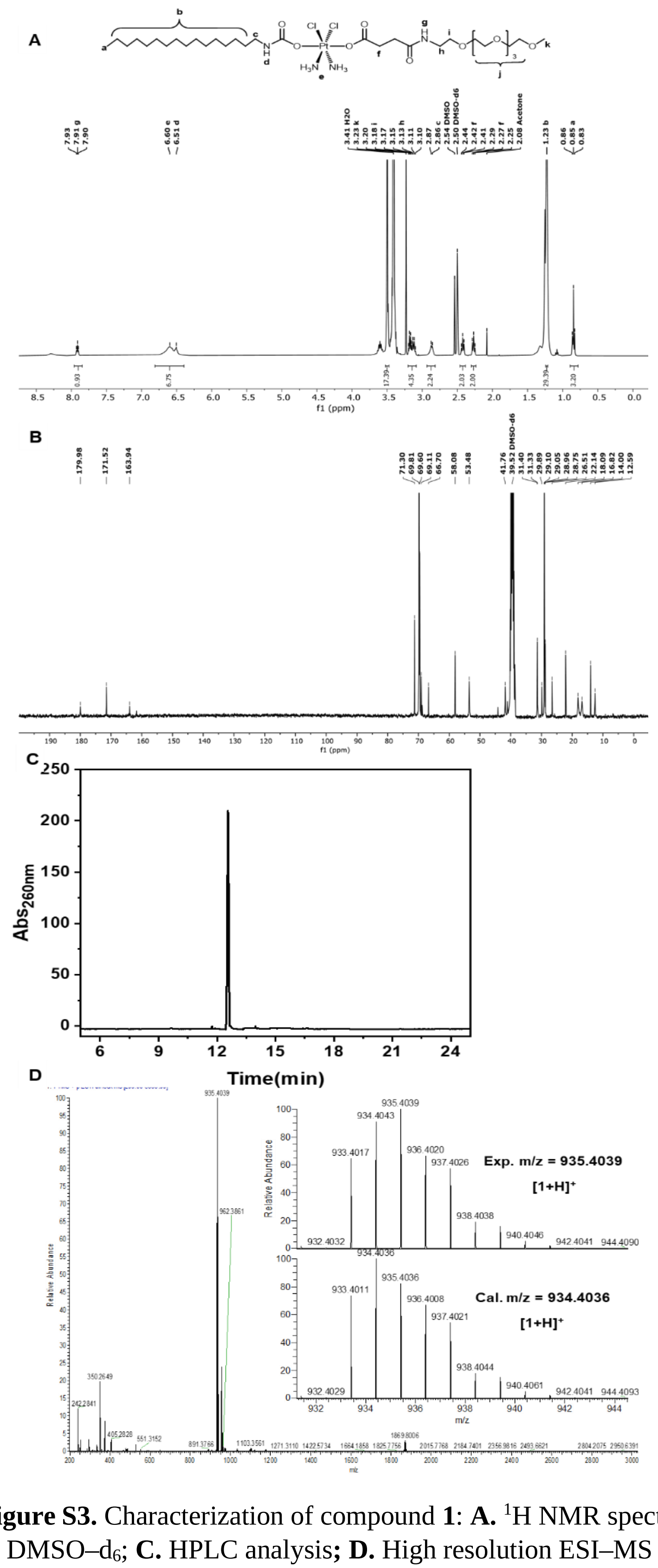

Figure S3. Characterization of compound **1**: **A.** ^{1}H NMR spectrum in DMSO–d_6; **B.** ^{13}C NMR spectrum in DMSO–d_6; **C.** HPLC analysis**; D.** High resolution ESI–MS spectra.

Supporting Information

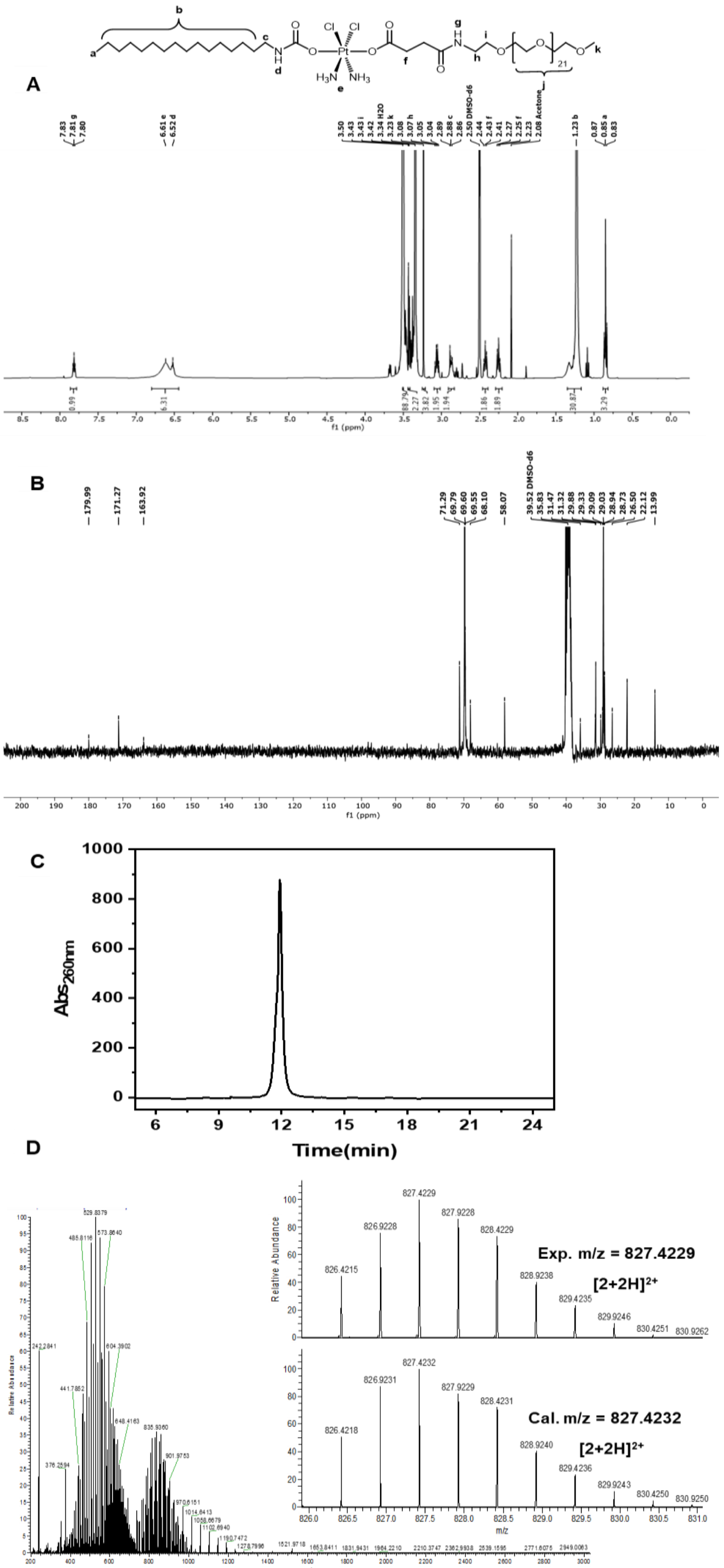

Figure S4. Characterization of compound **2: A.** ^{1}H NMR spectrum in DMSO–d$_6$; **B.** ^{13}C NMR spectrum in DMSO–d$_6$; **C.** HPLC analysis; **D.** High resolution ESI–MS spectra.

Supporting Information

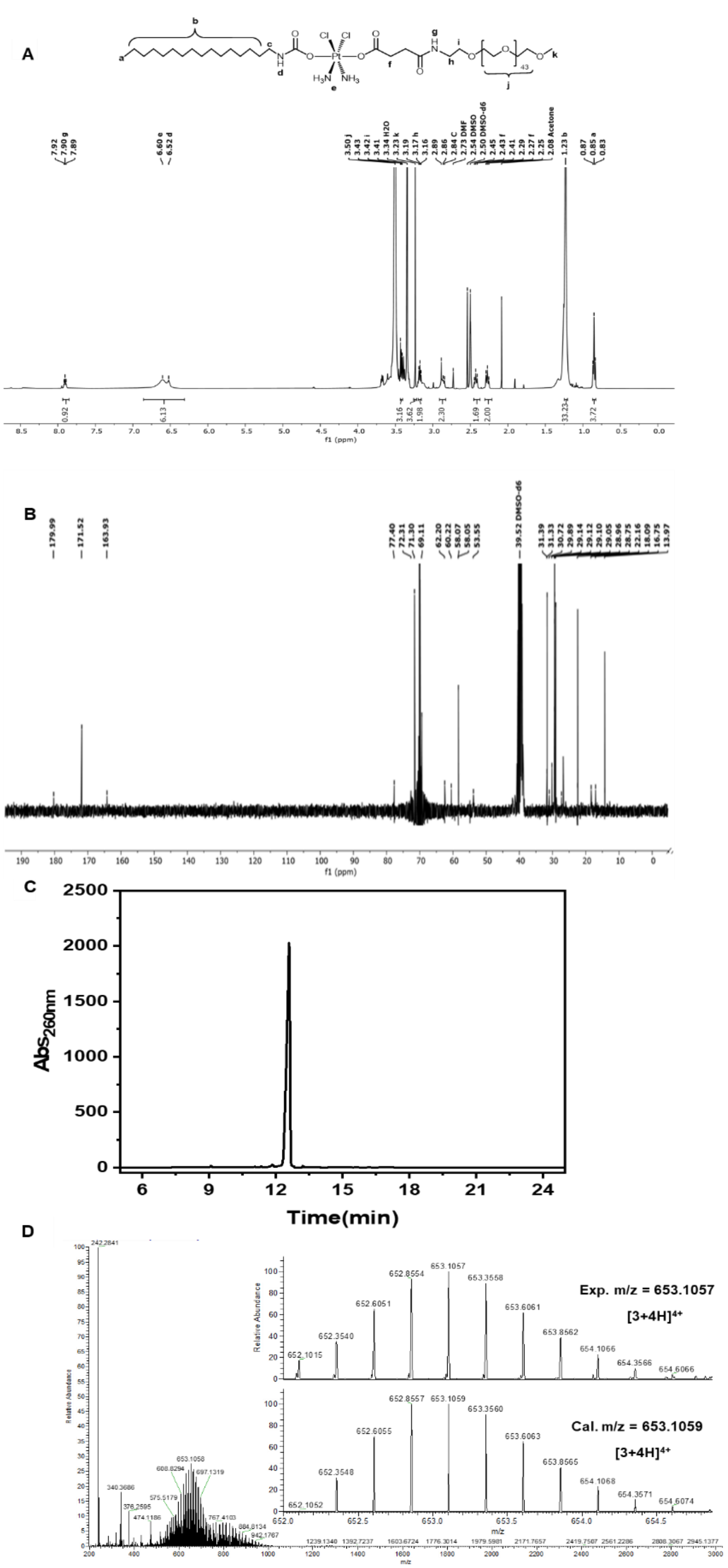

.

Figure S5. Characterization of compound **3: A.** ^{1}H NMR spectrum in DMSO–d_6; **B.** ^{13}C NMR spectrum in DMSO–d_6; **C.** HPLC analysis; **D.** High resolution ESI–MS spectra.

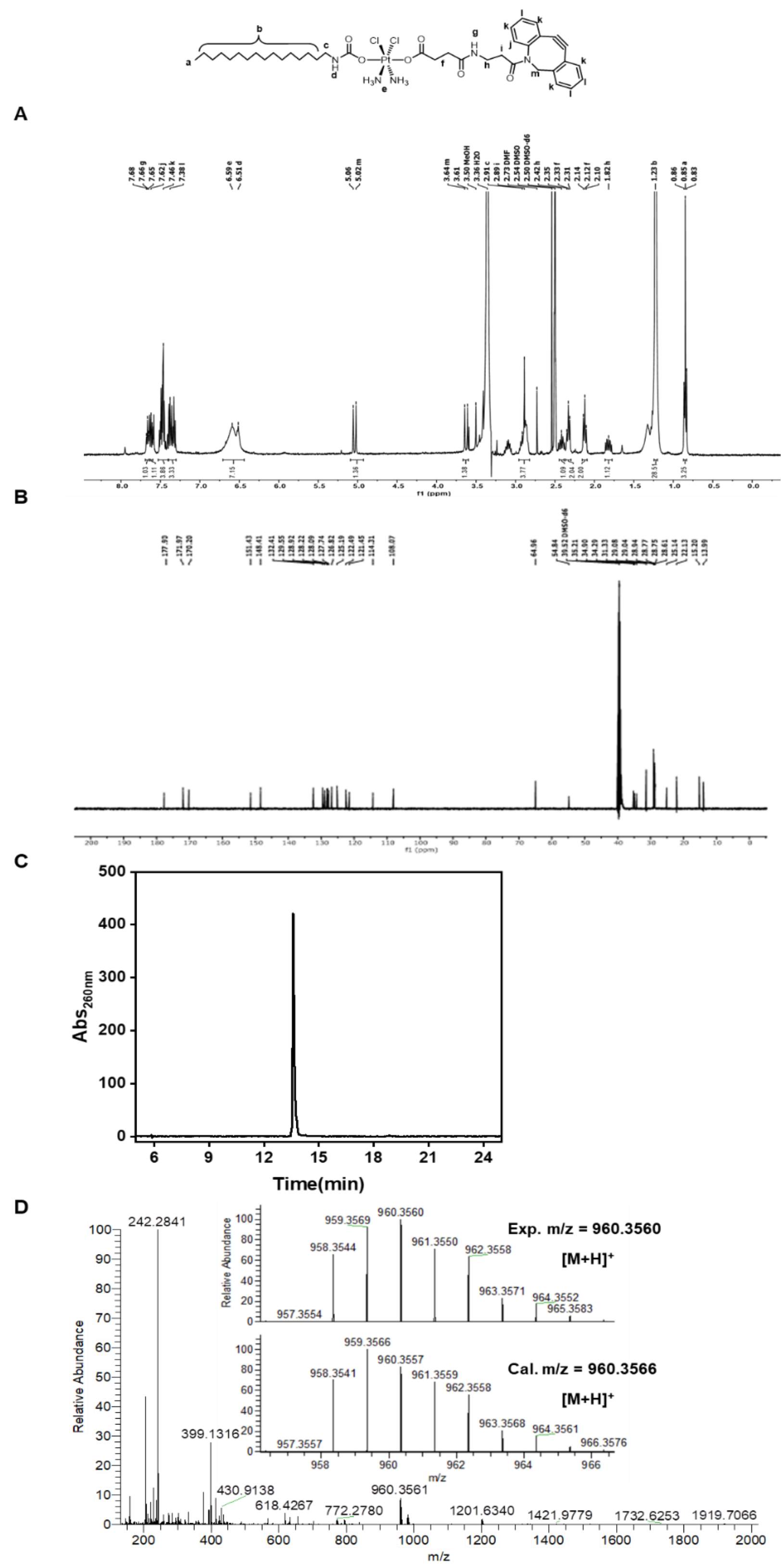

Figure S6. Characterization of compound **C16Pt–DBCO**: **A.** ^{1}H NMR spectrum in DMSO–d_6; **B.** ^{13}C NMR spectrum in DMSO–d_6; **C.** HPLC analysis; **D.** High resolution ESI–MS spectra.

A

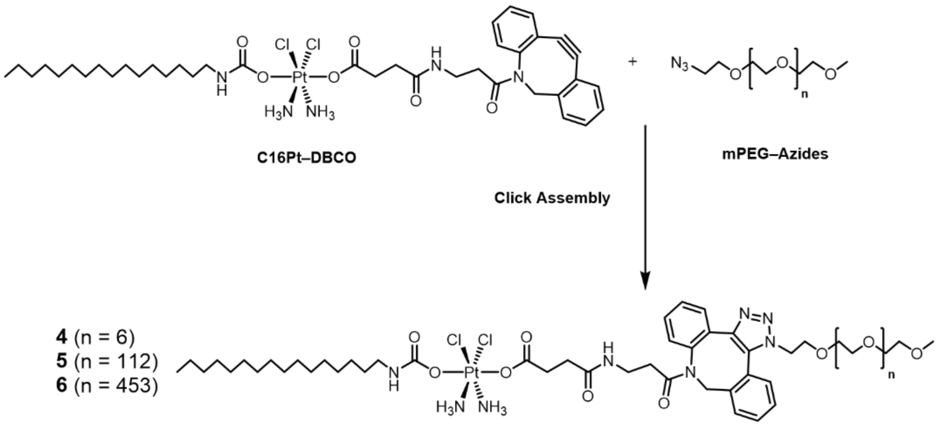

B

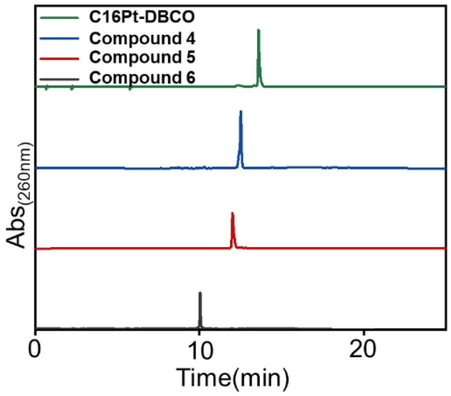

C

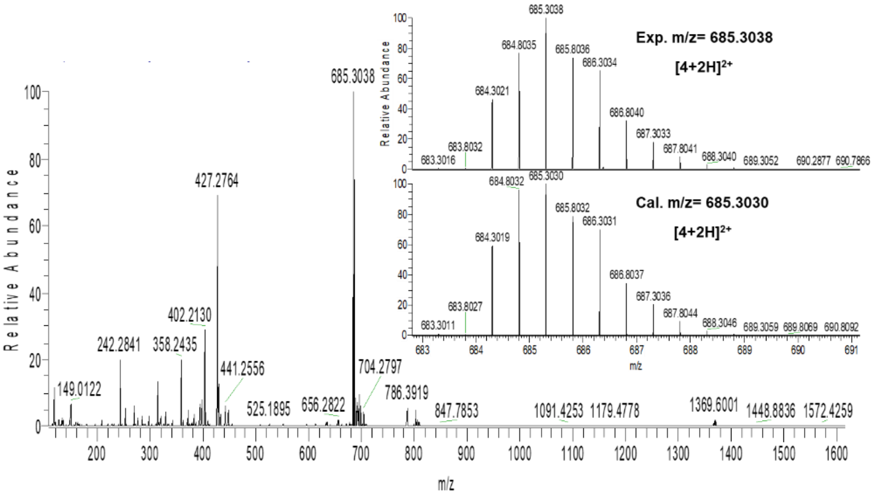

Figure S7. Characterization of compound **4–6**: **A**. Formation of Complex **4–6** via copper-free click reaction between **C16Pt–DBCO** and **mPEG–Azides**; **B**. HPLC Analysis of C16Pt-DBCO and **4–6**; **C**. High resolution ESI–MS spectrum of compound **4.**

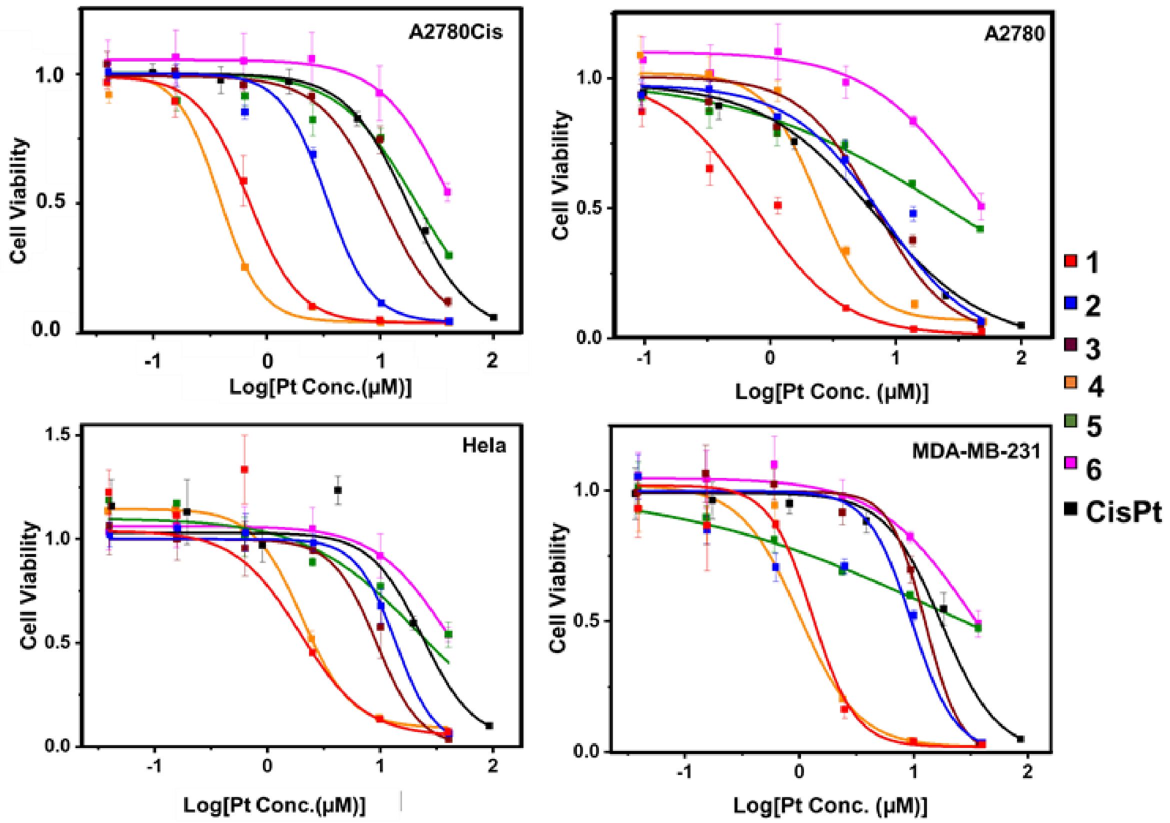

Figure S8. Killing curves of compounds **1**–**6** and cisplatin (CisPt) incubated for 48 hours using A2780cis, A2780, MDA–MB–231, and Hela cells.

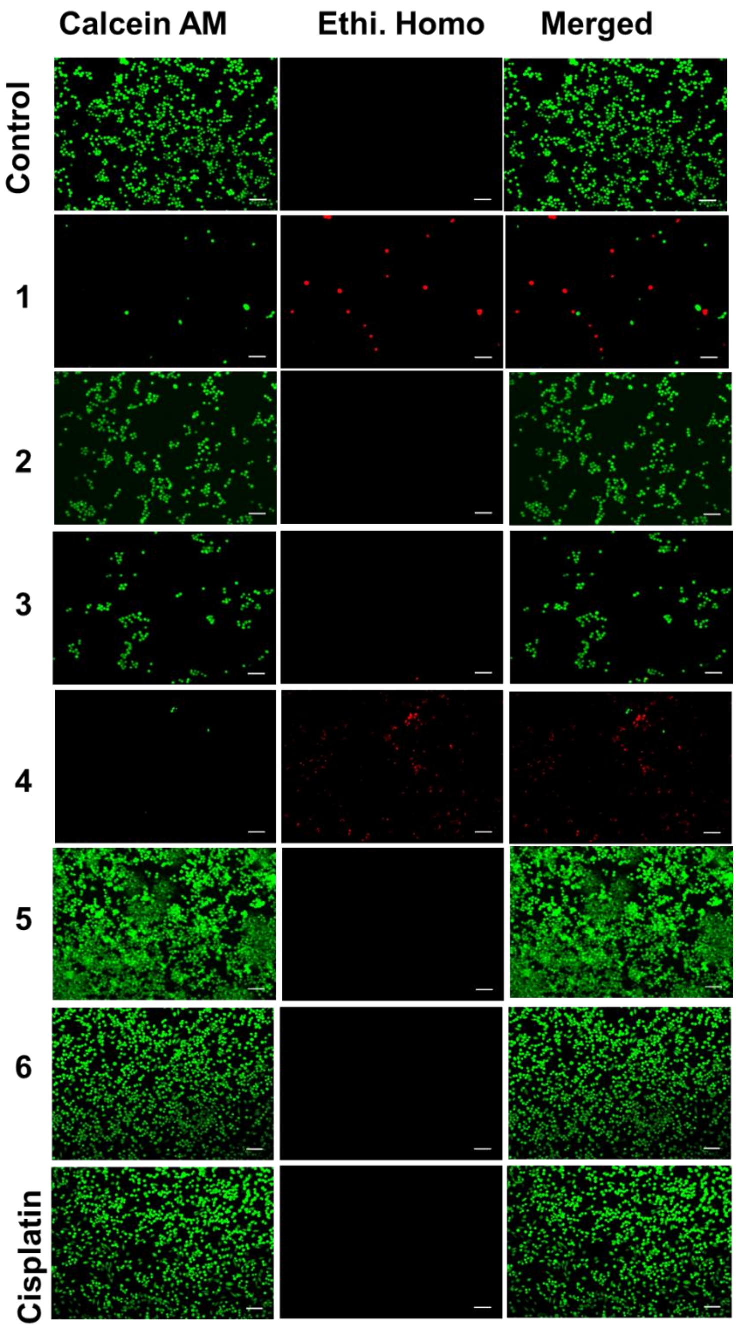

Figure S9. Fluorescence microscope images of **Live/Dead assay** for A2780cis cells treated with (Control), **1**–**6,** (1 µM, 48 h) and **cisplatin** (100 µM) at 37°C under 5% CO_2. Scale bar = 100 µm.
